# Comparison of internal vs. external vs. holistic attentional focus in golf putting performance among children: the role of skill level

**DOI:** 10.3389/fpsyg.2026.1801673

**Published:** 2026-05-11

**Authors:** Kaijun Wang, Dan Li, Can Feng, Chi-Lun Tsai

**Affiliations:** 1 Ice and Snow College, Jilin Sport University, Changchun, China; 2 College of Leisure and Social Sports, Jilin Sport University, Changchun, China; 3 Graduate Department, Jilin Sport University, Changchun, China; 4 Department of Sport Psychology, Sport Science Faculty, Leipzig University, Leipzig, Germany

**Keywords:** attentional, child athletes, golf, motor learning, performance

## Abstract

Attentional focus instructions reliably enhance motor performance in adults, yet little is known about whether these effects extend to children. This pilot study examined how internal, external, and holistic focus strategies influence golf putting performance in 10–12-year-old novice and beginner child golfers. Twenty-four boys (*M* age = 11.29 years) completed a within-subjects putting task across four conditions (control, internal, external, and holistic), with skill level as a between-subjects factor. Participants first completed a control block of 15 pretest putts and then three 15-putt experimental blocks under internal-, external-, and holistic-focus instructions. Before each attentional condition, participants rated anxiety, received standardized instructions, and completed five practice putts. All three attentional focus cues significantly improved putting accuracy compared with the control condition, *ps ≤* 0.003, whereas no differences emerged among the cue types, *ps* > 0.44. Beginner children performed better overall than novices, *p =* 0.007, but skill level did not moderate the effects of attentional focus, *p* = 0.084. Anxiety levels did not differ across conditions, *p* = 0.497, confirming that performance changes were not confounded by emotional responses. These findings suggest that attentional focus benefits children’s precision performance, but cue-type distinctions commonly observed in adults do not appear in late childhood. The results highlight the need for developmentally appropriate cue strategies in youth sport.

## 1 Introduction

Accumulating evidence shows that attentional focus instructions can meaningfully influence motor performance (Nicklas et al., 2024; Wang et al., 2021; Wulf et al., 2001; Wulf, 2013). Wulf et al. (2001) and Wulf and Shea (2002) demonstrated the advantages of adopting an external focus compared with an internal focus of attention when performing a motor task. An external focus is concentrating on the intended effect of the movement (e.g., focusing on the pendulum-like movement of the putter or the trajectory of the golf ball toward the hole). In contrast, an internal focus refers to concentrating on specific aspects of one’s own body movements (e.g., focusing on how the arms, wrists, or shoulders move during putting). It is also useful to distinguish between task-relevant and task-irrelevant attentional focus. In the present context, focusing on the putter head or ball trajectory would be task-relevant, whereas focusing on a cue unrelated to the control of the putting action would be task-irrelevant; this distinction may matter because relevant attentional strategies, especially externally relevant focus, have been linked to better-skilled performance than irrelevant strategies (Saemi et al., 2017). Previous studies have consistently shown that directing attention externally can facilitate more fluent and automatic motor control, whereas internal focus may introduce excessive conscious regulation and impair performance (Nicklas et al., 2024; Wulf and Lewthwaite, 2016). At the same time, recent review evidence has questioned whether external focus shows a consistently superior effect over internal focus across motor tasks, suggesting that this advantage may be less robust and less universal than earlier summaries implied (McKay et al., 2024).

In precision-based sports such as golf, the role of attentional focus may be particularly consequential because successful putting requires fine visuomotor coordination, controlled force production, and stable postural regulation under minimal movement variability (Schmidt and Lee, 1998; Vickers, 1992). Unlike cyclic or continuous tasks such as swimming or running, golf putting is a discrete, self-paced skill in which athletes must integrate visual information, proprioceptive cues, and kinaesthetic feedback to align the putter face, regulate swing amplitude, and produce an accurate ball trajectory (Hasegawa et al., 2020). Even small shifts in attentional allocation can disrupt movement fluidity, introduce compensatory muscle activation, or affect timing control, ultimately resulting in directional errors or distance misjudgment. Empirical studies in golf have shown that internal focus cues (e.g., “keep your wrists straight”) can interfere with automaticity and degrade putting accuracy, whereas external focus cues (e.g., “swing toward the target line”) generally improve consistency and reduce movement variability (Wulf et al., 1999). However, recent findings also suggest that alternative cueing strategies, such as holistic focus, may positively affect motor performance. A holistic focus, defined by Becker et al. (2019), directs attention to the general feeling of the movement or the sensations associated with the movement. It may also facilitate performance by reducing conscious control over specific movement components and encouraging a more global and automatic mode of movement execution. The holistic focus is an extension of an external focus, as it emphasizes the effect of the intended movement (Sadowski et al., 2024). For example, Becker et al. (2019) investigated the effects of a holistic focus of attention compared with an internal and external focus of attention in a simple motor task. They observed that the holistic focus of attention and external focus performed significantly better than the individual who adopted internal focus (Zhuravleva and Aiken, 2023). However, individuals who adopted holistic and external focuses did not perform significantly differently. More recent studies have also directly examined holistic focus across different skill levels and sport contexts. For example, Noroozi et al. (2024) found that both novice and skilled karatekas performed similarly under holistic and external focus conditions, and outperformed under the internal focus condition in a standing long jump task. In addition, Shooli et al. (2024) reported the benefits of external and holistic attentional cues over the internal focus in skilled basketball players. Nevertheless, most of this study has been conducted with adults or older adolescents in different skill levels, and there is comparatively little evidence on how attentional focus operates (internal-focus vs. external-focus vs. holistic-focus) in child golfers with different skill levels during complex motor tasks such as golf (Brocken et al., 2016).

Although a substantial body of evidence demonstrates that attentional focus strategies reliably enhance precision performance in adults, particularly in tasks such as golf putting that require stable visuomotor coordination and automatic control (Wulf, 2013), whether these findings generalize to children remain unclear. Prior studies suggest that age is a critical moderating factor in attentional focus effects, as children differ from adults in their cognitive control, perceptual integration, and movement automaticity (Diamond, 2013; Kong et al., 2025; Ludyga and Herrmann, 2025). These developmental differences raise questions about whether children can benefit from external or holistic focus cues to the same extent as adults, or whether they respond differently due to ongoing maturation of attentional regulation. Despite these theoretical implications, research specifically examining how children use attentional focus strategies during precision-based motor tasks is extremely limited, although one study directly compared internal, external, and holistic attentional focus instructions in children at two skill levels. Specifically, Saemi et al. (2023) examined experienced and novice children and found that an external focus was beneficial in experienced children, whereas no clear advantage of any attentional focus strategy was observed in novices. However, that study was conducted on a front-crawl swimming task, whereas golf putting is a self-paced precision task that requires performers to control movement initiation and execute a single accurate action toward a stationary target. Accordingly, the attentional and motor-control demands of golf may differ from those of continuous tasks, and a systematic review further highlighted that no studies to date have tested these effects in child golfers (Flôres et al., 2025). In addition, some scholars have demonstrated and argued that attentional focus effects are moderated by motor skill level, with skilled performers often benefiting more consistently from external focus, whereas less-skilled performers show more variable or task-dependent effects (Perkins-Ceccato et al., 2003; Toner and Moran, 2014). Given that motor skill acquisition and automaticity are still developing in childhood, it remains unclear whether this skill-dependent pattern generalizes to child athletes.

To address this gap, the present pilot study investigates whether internal, external, and holistic focus cues differentially influence putting performance in novice and beginner child golfers. By examining both skill levels and attentional focus conditions within a precision, self-paced task, this study evaluates whether patterns established in adults can be meaningfully extended to child athletes. Based on developmental evidence suggesting that children rely more on conscious motor control (Kong et al., 2025; Ludyga and Herrmann, 2025), the internal focus of attention may play a functional role in children’s motor performance. At this developmental stage, directing attention to movement-related cues may remain functional because conscious monitoring is still part of children’s motor regulation rather than a disruption to already automatized control. In addition, because anxiety may influence attentional allocation during motor performance, it was treated as a potential confounding factor rather than an experimental variable in the present study. Accordingly, participants’ anxiety was assessed using a visual analog scale (VAS), so that this factor could be controlled in the analyses. Therefore, this study hypothesized that the effects of attentional focus strategies on putting performance would not necessarily impair performance in either novice or beginner golfers, and that internal focus would produce effects comparable to those of external and holistic focus conditions (hypothesis 1). The findings may provide the first empirical evidence on how internal, external, and holistic attentional focus cues operate in child golfers during a complex motor task, thereby offering evidence-based guidance for coaching strategies in youth golf training.

## 2 Materials and methods

### 2.1 Participants

A total of 24 male child golfers were recruited through convenience sampling, including 12 male novice child golfers and 12 male beginner child golfers (Table 1). The study used a mixed-factorial design consisting of one within-subject factor (different attention conditions) and one between-subject factor (skill level). To ensure participants’ homogeneity and data quality, strict inclusion and exclusion criteria were applied.

**Table 1:** Descriptive statistics for participant characteristics by group.

Variable	Group	Mean	SD
Age	Novice golfers	11.08 yr	1.31
	Beginner golfers	11.5 yr	0.8
Height	Novice golfers	154.33 cm	4.75
	Beginner golfers	153.17 cm	3.76
Weight	Novice golfers	35.88 kg	3.27
	Beginner golfers	36.67 kg	2.27
Experience	Novice golfers	0 yr	0
	Beginner golfers	1.43 yr	0.43

SD = Standard deviation. Units are presented as years (yr), centimeters (cm), and kilograms (kg).

A sensitivity power analysis was conducted using G*Power 3.1.9.7 for a repeated-measures ANOVA with within–between interaction (*α* = 0.05, power = 0.80, two groups, four repeated measurements), indicating that the present sample size (*N* = 24) was sufficient to detect a medium-to-large effect size (*f* = 0.42). This estimated effect size is comparable to those reported in previous attentional focus and precision sport, supporting the adequacy of the existing sample for detecting meaningful effects (Nicklas et al., 2024).

Participants were eligible when they were right-handed, as verified using the Edinburgh Handedness Inventory (Oldfield, 1971), between 10 and 12 years old, had normal or corrected-to-normal vision, and demonstrated typical visual selective attention based on performance on the Trail Making–A Test (Partington and Leiter, 1949). Beginner child golfers were required to have accumulated at least 1 year of formal golf experience, whereas novice golfers had no prior golf experience. Participants were excluded when they reported a history of neurological or psychiatric conditions or the use of medication known to influence central nervous system functioning. All procedures conformed to the ethical standards outlined in the Declaration of Helsinki and were approved as specified by the Research Ethics Committee of Jilin Sport University.

### 2.2 Measures

#### 2.2.1 Golf putting task

Participants perform an indoor golf putting task on a laboratory-based artificial green (500 × 200 cm) equipped with a regulation-sized golf hole (diameter = 10.8 cm). The putting distance was set at 3 m, consistent with prior golf-putting research involving lower-skilled performers (Chiviacowsky et al., 2013), to provide a sufficiently challenging task without being overly difficult. Motor preparation time followed the operational definition proposed by Wang et al. (2020) —the interval between placing the putter behind the ball and initiating the backswing. The golf putting performance was measured using a tape measure.

#### 2.2.2 Visual analog scale (VAS) for anxiety

To ensure anxiety did not confound performance outcomes across attentional conditions, state anxiety was assessed using an 11-point Visual Analog Scale (VAS), anchored from 0 (not experiencing anxiety at all) to 11 (maximum possible anxiety level, following procedures consistent with Wang et al., 2023). Participants completed the VAS rating before the onset of each attentional condition.

### 2.3 Task and procedure

The experimental procedure followed the general framework of Perkins-Ceccato et al. (2003). Upon arrival, participants were informed of the study purpose and completed 10 warm-up putts. During this period, novice golfers additionally viewed a 5-min instructional video on basic putting techniques to ensure minimal task familiarity. Following warm-up, participants performed 15 pretest putts to establish baseline performance (control condition).

In this condition, no specific attentional focus instructions were provided. Participants then completed three 15-putt experimental blocks under internal-, external-, and holistic-focus instructions. The control condition was administered first, whereas the three attentional-focus conditions were counterbalanced across participants to control for learning effects. Participants were instructed to aim to place the ball as close as possible to the target on every trial. Before each attentional condition, participants rated their anxiety level using a visual analog scale (VAS). A 5-min seated rest was provided between conditions to minimize fatigue. Before each condition, participants received approximately 5 min of standardized instructions explaining the assigned attentional focus strategy. To ensure understanding and familiarization, participants performed five practice putts while applying the instructed strategy. The experimenter confirmed that participants understood the instructions and were ready to proceed before the formal testing trials began. No additional analogies were used; instead, the instructions were delivered in simple, age-appropriate language, and the experimenter confirmed participants’ understanding before testing. The attentional focus instructions were standardized as follows:

(a) Internal-focus condition: Direct attention to specific aspects of one’s own movement (Chinese: 将注意力放在自身动作的具体部位).
(b) External-focus condition: Focus attention on the putter head during execution (Chinese: 将注意力放在推杆头的运动上).
(c) Holistic-focus condition: Attend to the smooth, coordinated overall feel of the entire movement (Chinese: 专注于整体动作的流畅与协调感).

**Figure 1:**
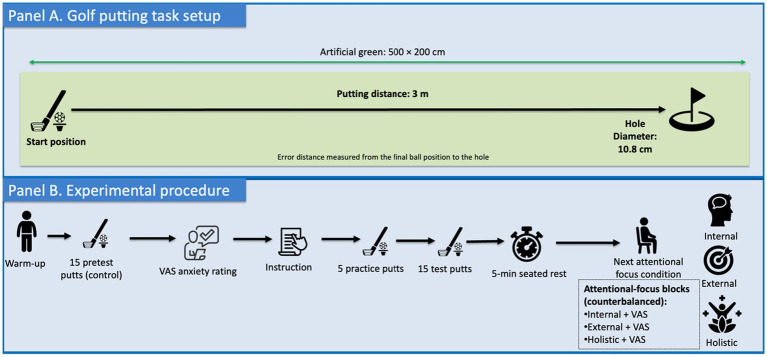
Schematic illustration of the golf putting task and procedure.

To minimize the learning effects, no performance feedback (knowledge of results) was provided at any point. To assess adherence, participants completed a brief yes/no manipulation check after each condition. A schematic illustration of the golf putting task setup and experimental procedure is shown in Figure 1.

### 2.4 Statistical analysis

All data were analyzed using SPSS version 26.0 (IBM). To evaluate whether anxiety differed across conditions or skill level, a 2 (Skill Level: beginner vs. novice) × 4 (Condition: control, internal-focus, external-focus, or holistic-focus) mixed-design two-way analysis of variance (ANOVA) was conducted on VAS anxiety scores.

To examine putting performance, a corresponding 2 × 4 mixed-design two-way ANOVA was conducted. When significant main effects or interactions were found, Bonferroni-adjusted *post hoc* comparisons were applied to control for Type I error inflation. The significance threshold for all analyses was set at *α* = 0.05.

## 3 Results

### 3.1 Manipulation check (anxiety level)

A 2 (Skill Level: Beginner vs. Novice) × 4 (Condition: Control, Internal-focus, External-focus, or Holistic-focus) mixed-model ANOVA revealed no significant main effect of Condition, *F* (3, 66) = 0.802, *p* = 0.497, and *η*_p_^2^ = 0.035, indicating that anxiety levels did not differ across the four focus conditions. Similarly, the Condition × Skill Level interaction was not significant, *F* (3, 66) = 0.367, *p* = 0.777, and *η*_p_^2^ = 0.016, suggesting that changes in anxiety across conditions were comparable for novices and beginner child golfers. For the between-subjects factor, no significant main effect of skill level was observed, *F* (1, 22) = 0.188, *p* = 0.669, and *η*_p_^2^ = 0.008. This indicates that novice and beginner child golfers reported similar overall anxiety levels.

### 3.2 Manipulation check (adherence to attentional focus instructions)

A brief self-report manipulation check was conducted to examine whether participants adhered to the instructed attentional focus strategy. After completing each attentional focus condition, participants were asked whether they had followed the given instructions during the trials and responded with either “yes” or “no.” The results indicated full adherence across all conditions. Specifically, 100% of participants reported following the instructions in the internal-focus condition (yes = 100%, no = 0%), the external-focus condition (yes = 100%, no = 0%), and the holistic-focus condition (yes = 100%, no = 0%).

### 3.3 Golf putting performance

**Figure 2:**
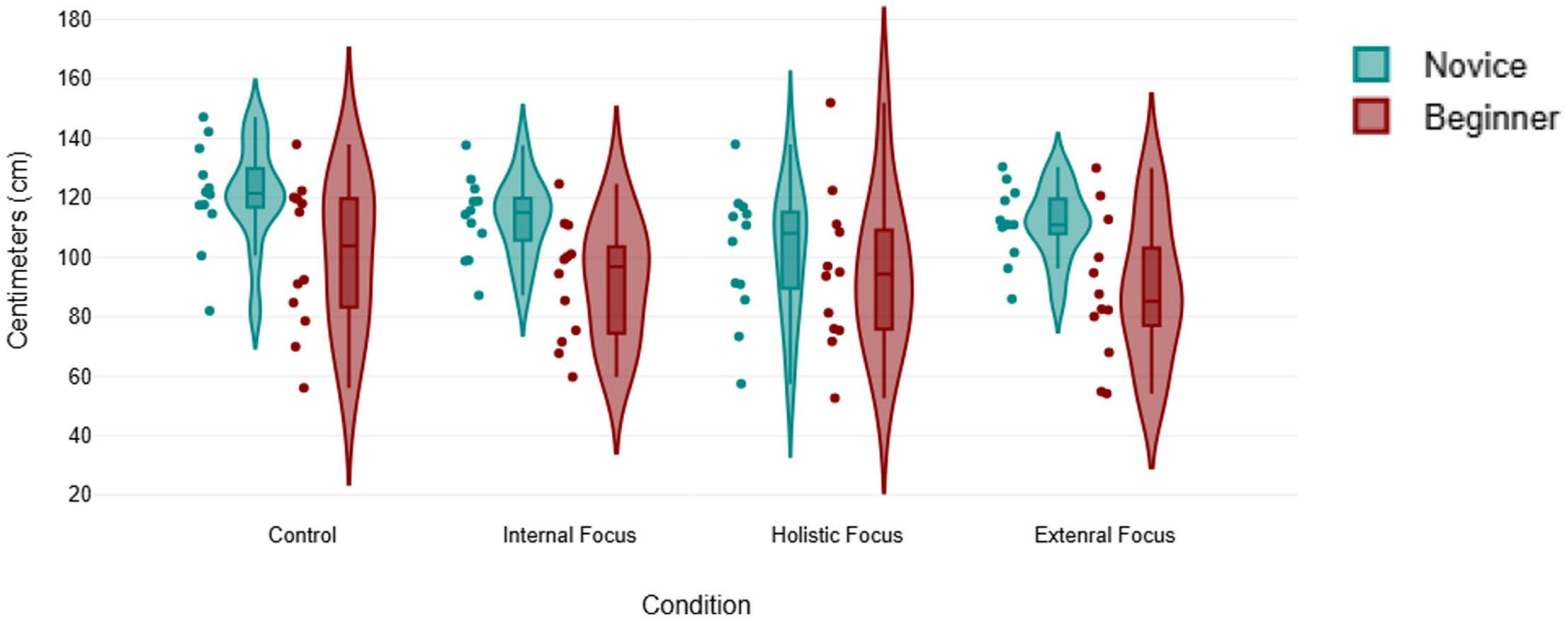
Violin plots depict golf putting performance (cm) for novice and beginner child golfers. Each violin illustrates the distribution of performance, with overlaid individual data points and internal boxplots showing the median and interquartile range. Beginner child golfers consistently demonstrated less error than novice golfers across all conditions, and both groups exhibited a similar performance pattern across focus conditions.

Golf putting performance across conditions and groups is presented in Table 2 and Figure 2. A mixed-model ANOVA revealed a significant main effect of Condition, *F* (3, 66) = 9.24, *p* < 0.001, and *η*_p_^2^ = 0.29, a significant main effect of Skill Level, *F* (1, 22) = 8.68, *p* = 0.007, and *η*_p_^2^ = 0.28. No significant Condition × Skill Level interaction was found, *F* (3, 66) = 0.30, *p* = 0.084, and *η*_p_^2^ = 0.013, indicating that novices and beginner child golfers showed similar patterns across conditions. The *post hoc* analyses indicated that participants exhibited longer distances from the target in the control condition compared with all three attentional-focus conditions. Specifically, errors in the control condition were significantly greater than in the internal-focus (mean difference = 8.29 and *p* = 0.001), external-focus (mean difference = 10.58 and *p* = 0.003), and holistic-focus conditions (mean difference = 14.04 and *p* < 0.001). These differences remained significant after the Bonferroni corrections. In contrast, no significant differences were observed among the internal, external, and holistic-focus conditions (*ps* > 0.44), suggesting that all three forms of focus cues similarly reduced error distance. Regarding skill level, beginner child golfers produced smaller distances from the target than novices overall (mean difference = 19.7 and *p* = 0.015), confirming the main effect of skill level.

**Table 2:** Descriptive statistics for golf putting (cm) across conditions by group.

Group	Control	Internal	Ext	Hol
Novice golfers	121.04 (± 17)	113.26 (± 13.6)	111.41 (± 12.5)	101.34 (± 22.3)
Beginner golfers	100.51 (± 25.1)	91.81 (± 19.9)	88.97 (± 24.1)	94.74 (± 26.5)
Total	110.77	102.53	100.19	98.04

± = Standard deviation. Int is the internal focus of attention, Ext is the external focus of attention, and Hol is the holistic focus.

## 4 Discussion

The present findings demonstrate that all three attentional focus cues (internal, external, and holistic) enhanced children’s golf putting performance relative to the control condition, yet no differences emerged among the three cue types. This pattern diverges from the robust external-focus advantage commonly observed in adults performing precision-based tasks such as golf putting (Bell and Hardy, 2009; Wulf, 2013). However, this interpretation should also be considered in light of recent review evidence suggesting that the superiority of external over internal focus is not consistently observed across the broader literature (McKay et al., 2024). Rather than representing a simple mismatch with adult findings, this discrepancy suggests that attentional focus operates under fundamentally different developmental constraints in childhood. Prior studies indicate that the benefits of external focus depend on the presence of highly proceduralized motor representations and mature neural pathways for automatic control (Wulf and Lewthwaite, 2016). Children, however, have not yet developed the stable motor schemas or neuromuscular efficiency required for the automaticity-based mechanisms that underlie the external focus effect. The absence of a skill-level moderation effect in the present study further reinforces this interpretation. Although beginner children showed better overall accuracy, their response patterns across cue types resembled those of novices, indicating that experience-related advantages in childhood do not yet approximate the expert–novice distinctions typically observed in adults.

The consistent superiority of all attentional focus cues over the control condition should not be interpreted as evidence that children discriminate meaningfully among cue types. Instead, the functional importance of attentional structuring during the early stages of motor development is highlighted. Children’s movement execution is heavily constrained by immature coordination, underdeveloped sensory integration, and limited attentional stability (Best, 2010; Best and Miller, 2010; Diamond, 2013). In self-paced precision tasks such as putting, the absence of a clear attentional frame—as in the control condition—may increase cognitive load and destabilize movement planning. Research on children’s perceptual–attentional systems indicates that they become particularly susceptible to distraction when attentional templates are not externally supported (Lavie et al., 2004). In this context, attentional structuring refers to directing attention toward task-relevant movement information, whereas preventing distraction refers to reducing attention to task-irrelevant thoughts or environmental stimuli. For example, a cue such as focusing on the putter head or on the smooth overall feel of the movement may not only guide children toward relevant aspects of task execution but also help prevent their attention from drifting to irrelevant internal thoughts or external distractions (Chen et al., 2022a; Wang et al., 2022b; Wu et al., 2023). From this perspective, attentional focus cues in childhood may function not only as performance-optimization tools but also as attentional scaffolding mechanisms that help organize perception and reduce interference. This explains why all three cue types improved performance and why their effects converged.

Adult models of attentional focus assume that external cues facilitate automatic control by reducing reliance on conscious monitoring. Yet this mechanism may not operate effectively in children. Recent studies further suggest that the effectiveness of external focus may depend on its temporal framing, with impact-focused external instructions outperforming throughout-swing external instructions in novice golf putting (Simpson et al., 2026). Developmental research shows that children rely heavily on proprioceptive and tactile information to regulate movement (Sigmundsson and Hopkins, 2010) and their neural systems are not yet capable of flexibly shifting between internal and external control modes (Diamond, 2013). As a result, even when adopting an external focus, children may continue to engage considerable proprioceptive monitoring, preventing the transition into automaticity typically observed in adults. Conversely, holistic cues may align naturally with children’s tendency to regulate movement based on global sensations rather than fine-grained, segmented analysis (Adolph and Franchak, 2017). Unlike the internal and external focus cues, which provide relatively clear attentional anchors, the holistic instruction may rely more on children’s subjective perception of movement quality. This may result in less consistent attentional engagement and strategy implementation across trials, which could help explain the wider variability observed in the holistic condition, particularly among the beginner group. This developmental alignment may explain why holistic focus produced improvements comparable to external and internal focus, and why no single attentional strategy was found to be superior in children.

Although beginner child golfers outperformed novices overall, both groups exhibited highly similar response patterns to the attentional focus manipulations. This suggests that skill level in the present sample influenced overall putting proficiency rather than the use of attentional focus cues. In other words, beginner children were more accurate overall, but they did not appear to derive a qualitatively different benefit from internal, external, or holistic cues than novices. This pattern differs somewhat from findings such as those of Saemi et al. (2023), in which experienced but not novice children benefited more clearly from external focus, and may reflect task-specific differences between swimming and golf putting. Recent studies on holistic focus suggest that relevant differences may also emerge at the level of movement processes rather than overt performance alone, as holistic focus has been linked to changes in kinematic and electromyography (EMG)-related outcomes in addition to behavioral effects (Becker and Avalos, 2024; Saemi et al., 2024). Accordingly, the similar putting outcomes observed for novice and beginner children in the present study do not rule out the possibility of underlying movement differences that were not captured by the present measures. In adults, attentional focus effects differ markedly between experts and novices due to experts’ more refined motor schemas and superior neural efficiency (Beilock et al., 2002; Chen et al., 2022b; Lu et al., 2025; Nicklas et al., 2024; Wang et al., 2020). However, developmental studies show that children do not acquire expert-like neural efficiency simply through additional practice (Crone and Richard Ridderinkhof, 2011). Childhood training primarily enhances gross coordination and rhythm but does not yet produce the proceduralized, automatized control observed in adult experts. This likely explains why skill level did not moderate attentional focus effects: both novice and beginner children operate within similar developmental boundaries, with neither group possessing the degree of motor automaticity required for differential sensitivity to cue types. Neurophysiological evidence further supports this interpretation. Adult athletes adopting an external focus show reduced prefrontal activation and greater sensorimotor efficiency, reflecting enhanced automaticity (Kal et al., 2013; Li and Smith, 2021). In contrast, children consistently display diffuse, non-specialized activation across frontal and sensorimotor areas during motor tasks (Caçola et al., 2018; Casey et al., 2005), suggesting immature neural organization. Such diffuse activation may reflect inefficient allocation of neural resources, such that children recruit broader control-related networks even for relatively simple motor performance. Under such developmental constraints, children are unlikely to exhibit the neural suppression of conscious control that underlies the external focus advantage in adults. As a result, they may be less able to shift from effortful, consciously monitored control to the more efficient and automatized processing mode (Cheng et al., 2024; Yu et al., 2025), which external focus is assumed to facilitate. Instead, all three cue types are processed in a similarly undifferentiated manner, leading to converging behavioral outcomes. This neurodevelopmental explanation provides a coherent account for why the expected differences among internal, external, and holistic focus were absent in the present study.

Taken together, the present findings show that attentional focus effects in child golfers differ meaningfully from adult patterns. While attentional focus cues reliably improved performance, no single cue emerged as superior. These results support recent arguments that external-focus recommendations derived from adult populations should not be assumed to generalize to youth sport (Pereira-Júnior and Bonuzzi, 2022). For children, the most effective coaching strategies may not involve selecting a single “optimal” focus type. Instead, they rely on providing cues that are developmentally appropriate, such as instructions that are simple, concrete, and structurally clear (Cordeiro-Costas et al., 2023). The present study highlights that attentional focus prescriptions for youth athletes must be grounded in developmental science rather than in direct extrapolation from adult motor behavior models.

### 4.1 Strengths and limitations

The present study has several strengths that add value to the existing literature. Most notably, this study extends attentional focus research to child golfers performing a self-paced precision task, a population and task context that has received little direct attention in previous studies (Flôres et al., 2025). In addition, by comparing internal, external, and holistic focus instructions within the same design and across two skill levels, the study provides a broader test of attentional focus effects in childhood beyond prior child-based studies conducted on other motor tasks (Saemi et al., 2023). Finally, the inclusion of a control condition, together with checks of anxiety and instruction adherence, strengthens confidence that the observed performance differences were attributable to attentional focus manipulation.

Despite these strengths, several limitations warrant consideration. First, because anxiety may influence attentional allocation during motor performance, it was treated as a potential confounding factor in the present study and assessed using a self-reported VAS. This allowed the study to account for anxiety at the measurement level; however, the use of self-report may not fully capture real-time fluctuations in anxiety during putting. Second, the absence of biomechanical or neurophysiological indices, such as electroencephalography (EEG), prevented examination of the underlying mechanisms responsible for the observed behavioral patterns. For example, previous studies have shown that several EEG components are associated with attentional processes involved in motor learning (Wang et al., 2024) and motor performance (Chen et al., 2019; Cheng et al., 2026; Li et al., 2025; Wang et al., 2022a; Wang et al., 2019; Yu et al., 2024). Third, although adherence was assessed after each condition, the brief yes/no self-report may not have captured the multidimensional nature of attentional focus implementation (see Kearney et al., 2025). Future studies should use more detailed manipulation checks. Finally, the sample was relatively small and homogeneous, consisting of boys of similar age and training backgrounds, which limits generalizability to girls, younger or older age groups, and wider developmental ranges. Given the possible developmental differences in fine motor skills between prepubescent boys and girls, this gender-specific sample should be considered a major limitation of the present study. Future studies with larger and more diverse samples are needed to confirm the stability and generalizability of the present findings. The present study also assessed only immediate performance and did not examine the long-term learning effects. These limitations also point to several directions for future research.

Future research should examine attentional focus effects across varying target distances, environmental constraints, and precision demands to determine whether external focus advantages emerge under more challenging conditions. Incorporating multimethod approaches, such as neurophysiological techniques, such as functional near infrared spectroscopy (fNIRS), biomechanical assessments, and movement-variability analyses, would provide deeper insight into how attentional cues influence children’s developing motor systems. Longitudinal designs may also clarify how attentional focus sensitivity evolves across childhood and adolescence (ages 6–8, 9–12, and 13–15 years) and whether children eventually converge toward adult-like patterns or follow distinct developmental trajectories. Such efforts may be essential for establishing the developmental boundaries of attentional focus theory and for informing evidence-based coaching practices in youth precision sports.

### 4.2 Practical implications

From a practical perspective, the present findings suggest that teaching and coaching golf putting in children should prioritize providing clear, simple, and developmentally appropriate attentional cues rather than selecting a single theoretically superior focus type. Because internal, external, and holistic cues all improved performance relative to the control condition, the practical value of attentional focus instructions in childhood may lie primarily in structuring children’s attention toward task-relevant information and reducing distraction, rather than in inducing the kind of movement automaticity typically proposed in adult performers. In applied settings, this implies that coaches and teachers may benefit from using brief, concrete, and age-appropriate cues delivered one at a time, while avoiding overly detailed technical explanations or multiple concurrent corrections that may exceed children’s attentional capacity. The absence of differential effects across cue types and skill levels further suggests that when working with child golfers, practitioners may flexibly select attentional cues according to the child’s comprehension, comfort, and immediate behavioral response, rather than assuming that one attentional focus strategy is universally optimal.

## 5 Conclusion

This study shows that while attentional focus cues reliably enhance golf putting performance in children, the cue-type differences widely reported in adults are absent in late childhood. The internal, external, and holistic cues were equally effective, indicating that children benefit more from receiving a clear attentional structure than from any specific focus direction. Although beginner child golfers outperformed novices overall, both groups responded similarly to the focus manipulations, suggesting that developmental constraints rather than skill level shape how attentional strategies operate during childhood. These findings caution against directly applying the adult-derived attentional focus guidelines to youth sport and highlight the importance of developmentally appropriate coaching practices. Future research should examine broader age ranges, incorporate biomechanical or neurophysiological measures, and clarify when cue-type distinctions begin to emerge during development.

## Data Availability

The datasets presented in this article are not publicly available due to ethical and privacy restrictions. Data may be provided by the authors upon reasonable request and subject to case-by-case consideration. Requests to access the datasets should be directed to the corresponding author.
